# The Training of Morphological Decomposition in Word Processing and Its Effects on Literacy Skills

**DOI:** 10.3389/fpsyg.2017.01583

**Published:** 2017-10-31

**Authors:** Irit Bar-Kochva, Marcus Hasselhorn

**Affiliations:** ^1^German Institute for International Educational Research, Frankfurt, Germany; ^2^Center for Individual Development and Adaptive Education of Children at Risk, Frankfurt, Germany; ^3^Edmond J. Safra Brain Research Center for the Study of Learning Disabilities, University of Haifa, Haifa, Israel; ^4^Department of Psychology, Goethe University Frankfurt, Frankfurt, Germany

**Keywords:** morphology, reading, spelling, literacy, intervention study, second language learners

## Abstract

This study set out to examine the effects of a morpheme-based training on reading and spelling in fifth and sixth graders (*N* = 47), who present poor literacy skills and speak German as a second language. A computerized training, consisting of a visual lexical decision task (comprising 2,880 items, presented in 12 sessions), was designed to encourage fast morphological analysis in word processing. The children were divided between two groups: the one underwent a morpheme-based training, in which word-stems of inflections and derivations were presented for a limited duration, while their pre- and suffixes remained on screen until response. Another group received a control training consisting of the same task, except that the duration of presentation of a non-morphological unit was restricted. In a Word Disruption Task, participants read words under three conditions: morphological separation (with symbols separating between the words’ morphemes), non-morphological separation (with symbols separating between non-morphological units of words), and no-separation (with symbols presented at the beginning and end of each word). The group receiving the morpheme-based program improved more than the control group in terms of word reading fluency in the morphological condition. The former group also presented similar word reading fluency after training in the morphological condition and in the no-separation condition, thereby suggesting that the morpheme-based training contributed to the integration of morphological decomposition into the process of word recognition. At the same time, both groups similarly improved in other measures of word reading fluency. With regard to spelling, the morpheme-based training group showed a larger improvement than the control group in spelling of trained items, and a unique improvement in spelling of untrained items (untrained word-stems integrated into trained pre- and suffixes). The results further suggest some contribution of the morpheme-based training to performance in a standardized spelling task. The morpheme-based training did not, however, show any unique effect on comprehension. These results suggest that the morpheme-based training is effective in enhancing some basic literacy skill in the population examined, i.e., morphological analysis in word processing and the access to orthographic representations in spelling, with no specific effects on reading fluency and comprehension.

## Introduction

The role of morphological processing in lexical access has been repeatedly shown in studies of different languages and orthographies (e.g., Drews and Zwitserlood, 1995; Clahsen, 1999; Diependaele et al., 2005; Frost et al., 2005; Penke, 2006; Smolka et al., 2007; Beyersmann et al., 2012). Morphemes are the smallest linguistic unit to convey a semantic meaning in a word. Morphologically related words share phonemes, graphemes and often also semantics, and therefore provide consistencies of recurring connections between spelling, sound, and meaning. Consequently, morphological decomposition of words should allow benefiting from these consistencies in reading and writing.

And indeed, studies of skilled readers indicate that morphological decomposition is an integral part of word processing. Methods addressing this aspect usually apply different morphological manipulations on the presentation of printed material. Sonnleitner (2013), for example, examined the role of morphological processing in reading of German by comparing the reading efficiency of pseudowords composed of real morphemes and of pseudowords composed of pseudo-morphemes. Children read the pseudo-words, which were built out of real morphemes faster than the pseudo-words built out of pseudo-morphemes. Another task used in this study was the Word Disruption Task, in which words were presented under three conditions: (1) the morphemes of each word were separated by a symbol (e.g., fang#en). (2) The syllables of each word were separated by a symbol (e.g., fan#gen). (3) The sequence of letters of each word was not violated by the symbol (e.g., #fangen). Children read the words separated by their morphemes significantly faster than the words separated by their syllables, and reading of the first was only marginally slower than reading under the no-separation condition. While taking into account that the separation of the syllables violated the sequence of letters of the words’ morphemes, and that this condition produced the largest disruption in reading rate, these results were taken to suggest that the processing of morphemes plays a role in word recognition.

One more widely used method in the study of morphological processing in word recognition is the priming task, in which visually (e.g., Smolka et al., 2007) or acoustically (e.g., Sonnenstuhl et al., 1999) presented prime words precede a visual presentation of target words in a computerized lexical decision task. In these studies, morphological relations between the primes and targets were found to accelerate the response to the targets. The extraction of the shared morpheme between the prime and the target was suggested to explain this effect. This was found even when primes were presented for a very brief duration (40–60 ms), which usually does not allow their conscious processing. In these studies, readers were found to extract the main morphological units, which carry the core meaning of words (word-stems or roots) from morphologically complex words (e.g., Frost et al., 2005; Marslen-Wilson et al., 2008). As the brief presentation duration of the primes is thought to capture initial processes of lexical processing (e.g., Rastle et al., 2000; Frost et al., 2005 see review by Rueckl, 2010), these results were taken to suggest that morphological analysis occurs at a very early stage of visual word recognition (Rastle et al., 2000; Frost et al., 2005; Marslen-Wilson et al., 2008; Rueckl, 2010). Consequently, morphological analysis was suggested to constitute a first priority in word processing (Marslen-Wilson et al., 2008).

However, findings are less consistent in studies of reading disabled participants (e.g., Elbro and Arnbak, 1996; Leikin and Hagit, 2006; Verhoeven and Schreuder, 2011; Quemart and Casalis, 2013; but see Deacon et al., 2006; Schiff and Raveh, 2007). While some studies suggest that these do not apply morphological analysis in visual word recognition (Raveh and Schiff, 2008), others suggest that poor readers even rely on morphological information as a strategy compensating for their word reading difficulties (Elbro and Arnbak, 1996; Burani et al., 2008). Nonetheless, the fact that morphological decomposition in visual word recognition was more consistently found in typical readers than in reading disabled participants may suggest that morphological analysis is not carried out as effectively or to the same extent by reading disabled participants as it is carried out by typical readers. Other studies further indicate that children with reading and language disabilities show deficient morphological awareness compared to age-matched typical readers (Carlisle, 1987; Fowler and Liberman, 1995; Casalis et al., 2004; Siegel, 2008). As previously suggested, interventions focusing on morphological skills may be particularly important for students struggling with literacy acquisition, as despite difficulty with some aspects of morphological processing, they may have the potential of addressing the morphological skills they do possess as a supporting strategy of reading (Goodwin and Ahn, 2010). With this in mind, morphological training might be regarded as an effective component of interventions aimed at enhancing literacy skills.

Studies on morphological instruction have indeed shown positive effects on literacy skills, and in speakers of different languages (Elbro and Arnbak, 1996; Nunes et al., 2003; Reed, 2008; Tsesmeli and Seymour, 2009; Bowers et al., 2010; Tsesmeli et al., 2011; Goodwin and Ahn, 2013). A meta-analysis of morphological intervention studies carried out in English-speaking school-aged children, who represent a general population of students, indicated a medium overall effect of morphological instruction on measures of literacy achievement (Goodwin and Ahn, 2013). Intervention effects were, however, moderated by the type of literacy skill tested, with significant and medium effects on morphological knowledge, phonological awareness, vocabulary, decoding and spelling, but not on reading fluency and comprehension. In another meta-analysis of these researchers, which focused on morphological instruction in students with difficulties in acquiring literacy skills, significant medium mean effect sizes were found for morphological interventions on phonological awareness, morphological awareness and vocabulary, and more modest effects on spelling and comprehension (Goodwin and Ahn, 2010). Morphological instruction was particularly effective for children with speech and language delay followed by English language learners, struggling readers, children with learning disabilities, and children with a reading disability.

The purpose of the present study was to examine the effect of a morpheme-based training on literacy performance, while adding to previous studies on morphological interventions in three respects: in the type of intervention provided, in the language and orthography examined, and in the characteristics of the population in focus. As far as the first respect is concerned, previously studied interventions focused on teaching participants explicit morphological rules and strategies. These provided, for example, instruction on morphological components and rules for combining them into words, instruction on linking morphemes to their grammatical functions, and introduction of word families and of strategies for identifying words by analogies (Goodwin and Ahn, 2010). While such explicit morphological knowledge is expected to contribute to various conscious processes involved in literacy skills (e.g., inferring the meaning of unfamiliar words in reading based on familiarity with morphologically related words), it may not necessarily influence implicit morphological processing involved in reading and writing. Fast morphological decomposition in word processing was, however, suggested to be an implicit process, which readers carry out automatically and without being able to report on it (e.g., Frost et al., 2005). Considering the important role suggested for this procedure in lexical access (e.g., Rueckl, 2010), the present study was designed to directly train it. This was done by developing a morpheme-based training program, which imposes a demand of quickly extracting the core morphological units from visually presented morphologically complex words. The program consisted of a lexical decision task, in which inflections and derivations were presented one after the other. In order to encourage the fast extraction of the core morphological unit of each word, the duration of presentation of the word-stems was restricted, while the pre- and suffixes remained on screen until response. The participants were tested for different literacy skills before training, right after training, and 1 month following training. The morpheme-based training was contrasted with a control training, which was designed to isolate the possible effect of the morphological manipulation. As the morpheme-based training included not only a morphological manipulation but also the imposing of time constraints on word processing –which in themselves were found to have positive effects on reading (Breznitz, 2006), the control training consisted of the same training program, except that the duration of presentation of a non-morphological unit was restricted.

An additional aspect referring to the type of intervention provided is that many of the previous studies applied several strategies of morphological instruction (Goodwin and Ahn, 2010, 2013). Consequently, the effect of each training technique is difficult to disentangle, which may be one reason for the great variability in the reported effects of morphological interventions. In contrast, the training examined in the current study focused on a single procedure of fast morphological decomposition in word processing. Finally, it may also be noted that morphological interventions are usually provided using explicit teaching, which necessitate the presence of a tutor. The training program examined here, however, was designed as a computer task, which allows independent work of the trainees.

The second addition of the current study refers to the language and orthography tested. In the present study, the effect of the morpheme-based training was examined in the German language. A Hebrew version of the same morpheme-based training was recently tested in the framework of a pilot examination among Hebrew speakers with a reading disability (Bar-Kochva, 2016). The results suggested an improvement in some basic literacy measures (spelling and accuracy in reading) of the morpheme-based training compared to a control intervention. However, the Hebrew language (with its Semitic origin), differs from the German one (with its Indo-European origin) in many aspects. One major difference lies in the morphological structure of the two languages, which may influence the salience and role of morphological processes in lexical access (Frost et al., 2005). Consequently, the effect of the suggested morpheme-based training on readers and spellers of the German language deserves a separate examination. Notably, the implications of differences between languages and orthographies on effects of training have received little attention in studies on literacy skills. Although the present investigation does not offer a direct comparison between languages and orthographies, it may provide some indication on whether a morphological training found to be relevant to the Hebrew language is also relevant to the German language.

Finally, the current study adds to previous intervention studies by focusing on a group of children struggling with literacy skills, who also have a migration background. The vast majority of studies on reading and writing addressed participants with a reading disability or readers with other developmental disabilities (e.g., language deficits), who usually speak the language of instruction as their first language. Until recently, reading disability was diagnosed when significant difficulties in accuracy and fluency in word recognition, decoding and/or spelling, were identified (DSM IV; American Psychiatric Association, 1994). Most recently, however, reading disability has been regarded as one aspect of a more general learning disability, while difficulties in reading comprehension and performance in other academic fields (e.g., mathematics) are also diagnostic criteria (DSM V; American Psychiatric Association, 2013). However, having a migration background, defined when a student or his or her parents were born outside the country of residence, has also been related to difficulties in acquiring literacy skills, and to difficulties in reading comprehension in particular (OECD, 2001, 2003, also see Aarts and Verhoeven, 1999; Verhoeven, 2000; but see Lesaux et al., 2007). It is important to note, that a migration background in itself may not necessarily be a source of difficulties in acquiring literacy skills, with the context of learning possibly having a mediating impact. For instance, the Programme for International Student Assessment [PISA] (OECD, 2001, 2003) indicated that in some countries (e.g., Germany and Belgium) adolescents with migration background reached literacy levels (defined in terms of reading comprehension) well below those of native students, and that these differences were more moderate in others countries (e.g., France, Sweden, United States, while having a migration background had very little effects in Australia and Canada). Lesaux et al. (2007) even found similar literacy skills in children with and without migration background in a longitudinal study of younger students (kindergarten to grade 4) in Canada. Therefore, the exact source of difficulty of the group in focus in the current study is difficult to trace. Their literacy deficits may lie in a developmental learning disability, and/or be related to their migration background, to the context of learning as well as to other possible factors [such as public policies relating to the filtering of immigrants by economic or educational background, see the Programme for International Student Assessment [PISA] (OECD, 2001, 2003)]. Despite the possible heterogeneity of this group, there is a practical need to search for intervention tools addressing the difficulties of these students, with their growing representation in different countries in the world. From a theoretical perspective, extending the examination of morphological training to these students may further contribute to the understanding of the role of morphology in acquiring literacy skills in various population.

Notably, of the various aspects involved in having a migration background (e.g., socio-economic status, parents’ level of education) an exposure to a language at home, which is not the language of instruction in school, appears to be a critical factor. In most countries surveyed in the Programme for International Student Assessment [PISA] (OECD, 2001, 2003), speaking another language at home raised by two to two and a half times the likelihood to be among the 25% of lowest achievers in reading literacy measures. In further analyses of the Programme for International Student Assessment [PISA] (OECD, 2003), it was found that when controlling for the language spoken at home, the performance gaps between students with and without migration background were substantially reduced in both mathematics and reading (also see Marx and Stanat, 2012). As previously formulated, while bilingualism in itself is not suggested to be a source of difficulty in acquiring literacy skills, students with migration background might be at a disadvantage if they lack opportunities to learn basic literacy skills in both of their languages (Marx and Stanat, 2012). Interventions approaching literacy through training of basic language skills were therefore suggested to address the needs of this population (Verhoeven, 1990; OECD, 2001, 2003; Marx and Stanat, 2012). Notably, the group in focus in this study may vary from certain groups of bilinguals who, for example speak the language of instruction with at least one of their parents. Nevertheless, previous findings indicating some disadvantage of bi-lingual children in language skills related to literacy acquisition, such as in vocabulary, lexical access and morpho-syntactic knowledge in their L2 (da Fontoura and Siegel, 1995; Hutchinson et al., 2003; Lesaux et al., 2006; Bialystok et al., 2008) may further support a linguistic approach to literacy intervention in children with poor literacy skills, who speak the language of instruction as their second language.

## Research Question and Hypotheses

The following research question was examined: what are the effects of a morpheme-based training, designed to train fast morphological analysis in word processing, on different literacy skills in a group of children struggling with literacy acquisition, and to whom the language of instruction is not their mother tongue. The hypotheses were as follows: (1) considering the important role found for morphological analysis in word processing, a general positive effect of the morpheme-based training was expected on literacy performance. (2) At the same time, in line with previous studies on morphological interventions (Goodwin and Ahn, 2010, 2013), including the study of the same morpheme-based training in Hebrew readers (Bar-Kochva, 2016), we expected to find effects of the program on basic literacy skills (on morphological analysis in word recognition and on spelling) more than on reading fluency and comprehension.

## Materials and Methods

### Participants

Children in the fifth and sixth grades (*N* = 47, mean age = 11.24 years, *SD* = 0.931) with migration background were tested. The children were recruited from two schools situated in middle-low to middle class neighborhoods in the area of Frankfurt am Main, Germany. All participants had a word reading efficiency score at the lowest 30th percentile in a standardized reading test (Salzburger Lese- und Rechtschreibtest, SLRT II; Moll and Landerl, 2010), and a non-verbal IQ score of above 75 (Zahlen-Verbindungs-Test, ZVT; Oswald and Roth, 1997). Considering that efficient word recognition is the building block of skilled reading (Lyon et al., 2003; Fletcher, 2009), the measure of word reading fluency was taken as a defining criterion. As general ability has a wide influence on various cognitive tasks, variance in the literacy skills resulting from differences in general ability was controlled by excluding from analysis students who performed below the average range in the non-verbal IQ task (ZVT; Oswald and Roth, 1997).

In line with the definition applied at the PISA surveys (e.g., OECD, 2001), the inclusion criterion for having a migration background was that the children were either born in Germany into families where both caregivers grew up in another country, or the children came themselves to Germany. In order to avoid possible effects of limited knowledge of German, participants who were not born in Germany were included in analysis only if they came to Germany as infants and were involved in a German speaking educational system from the age of 3 at the latest. Countries of origin were Turkey, Greece, Poland, Russia, Croatia, Portugal, Ethiopia, Ghana, Morocco, Afghanistan, and Iran. In addition, all children were exposed to a language other than German at home. This information was collected using a questionnaire referring to the following aspects: country of origin of the child and of each of his or her parents, the language first acquired by the child, age of acquisition of the German language and the dominant language spoken with each caregiver. All participants were exposed to a mother tongue other than German from birth on. Most children from all origins were exposed to German between birth and their first year of life, while eight children (whose origin was either from Turkey, Morocco, Ethiopia, or Afghanistan) started to learn German between the ages of two and three.

Participants were divided between two training groups: the one received the morpheme-based training (*n* = 24, 14 boys), and the other received a control training (*n* = 23, 11 boys). The assignment of participants into one of the two training groups was random. The study was approved by the local ethic committee of the German Institute for International Educational Research (DIPF), and participants provided written informed consent from their parent to take part in the study.

### Materials

#### Background Measures

Considering that, general ability has an influence on almost any cognitive task, and in order to verify similar general ability of the two groups, two tests were administered as approximations for non-verbal and verbal general abilities. The first was the “ZVT” (a number-connecting test. Oswald and Roth, 1997), which is a standardized test of cognitive processing speed, in which participants are required to link as fast as they can between visually presented numbers according to their order. The test is administered individually in a pencil and paper form. The test norms allow converting the mean cognitive processing speed into an IQ estimation. Internal consistency and 6-month test–retest reliability are between 0.84 and 0.98. The second test was the Vocabulary subtest from the German version of the WISC-IV (HAWIK IV; Petermann and Petermann, 2010), in which children are first presented with pictures and required to name them, and then asked to define words of various semantic categories (e.g., nouns, verbs, and adjectives). The test was administered individually.

Another background measure, which was administered in order to verify similar skills of the two groups, was the SLRT II decoding test (Moll and Landerl, 2010). The test is administered individually, while the items are presented on a printed page. The test comprises a list of 156 pseudowords, preceded by eight example items. The pseudowords were constructed based on legal structures in the language. The items in the test are arranged in columns, increasing in length and complexity. The children were instructed to read as fast and as accurately as they could and were stopped after one minute. Each correct reading of a pseudoword earned one point. Parallel test reliability coefficients of this test is above 0.90.

#### Morphological Analysis in Word Recognition

A Word Disruption Task was administered, which was a variation of the task previously used by Sonnleitner (2013). Three word lists were compiled. There were 29 morphologically transparent past participles of regularly inflected verbs in each list (German participles including the *ge*- prefix, the *-t* suffix and a base form in between, e.g., *getanzt*, meaning “danced”). The items were words previously used by Smolka et al. (2007) in their study (see Appendix B in their article). The mean participle frequency of each list was 4.10, 5.55, and 6 (as indicated by the CELEX database, Baayen et al., 1993, see Smolka et al., 2007), and the mean word length was 8.03, 8.24, and 8.34 letters. It should be noted, that there is no consensus on whether all German complex words are morphologically decomposed in reading (Clahsen, 1999; Penke, 2006; Smolka et al., 2007). Consequently, the words included in the lists were only of a class of words, which were repeatedly found to be analyzed into morphological units in reading of typical readers of German.

The task was administered individually. In each session, participants were presented with three word lists printed on three separate pages, which they were asked to read out aloud. Each list was presented under one of the following three conditions: (1) A no-separation condition. Words were presented with two identical non-orthographic symbols attached to their beginning and end (i.e., the symbols did not disrupt the sequence of letters in the words, e.g., #getanzt#, meaning “danced”). (2) A morphological separation condition. The pre- and suffix were separated by the non-orthographic symbols from the word-stem (ge#kauf#t, meaning “bought”). (3) A non-morphological separation condition. The symbols separated the words into three orthographic units, which did not convey a meaning (get#räu#mt, meaning “dreamt”), thereby violating the sequence of the morphemes’ letters. In trying to isolate the morphological factor in this task, and in order to reduce possible effects of visual differences between the morphological separation condition and this condition, the two symbols were integrated into the words in a location, which was as close as possible to their location in the morphological separation condition. Notably, integrating the symbols according to a different linguistic principle (such as between phonemes or syllables) would have created a visual difference between the morphological condition and the non-morphological condition (as more than two symbols would have been integrated into at least some of the words). Therefore, the segmentation of the non-morphological condition was guided by the location of the symbols, which created a random segmentation as far as the linguistic structure of the words is concerned.

Reading performance in the first condition was taken as a base-line measure of word reading fluency, to which reading under the two other conditions was compared. Similar reading proficiency in the no-separation condition and the morphological-condition would suggest that morphological analysis is integrated into the reading routine. Reduced word reading fluency in the morphological condition compared to the no-separation condition would suggest that morphological analysis is not carried out as part of the reader’s word recognition routine. The non-morphological separation condition was taken as a control condition, in order to examine whether the violation of the sequence of letters of the morphemes in each word created a disruption in word reading rate.

Each of the three word-lists appeared in all of these conditions across three testing times (administered before and after training, see **Table 1**). For example, in one session with a child, a specific word appeared in the no-separation condition, #getanzt#; in the second session, the same word appeared under the morphological-separation condition, ge#tanz#t; and in the third session it appeared under the non-morphological condition, get#an#zt. This procedure created nine versions of presentation altogether (three word lists × three presentation conditions). In order to avoid effects of order of administration, the lists were presented in a counterbalanced manner across participants, conditions and testing times. Participants were instructed to read as fast and as accurately as they can, and to ignore the symbols they see on the page. A measure of word reading fluency, which was based on the number of words correctly read within one minute, was calculated for each condition. In addition, the difference between the word reading fluency score in the no-separation condition and the morphological condition, and between the no-separation condition and the non-morphological condition was calculated. This measure served to estimate whether the location of the symbols violating the sequence of letters interfered in the process of word recognition (i.e., large differences would indicate a large disruption).

**Table 1:** An example of words presented in the Word Disruption Task in the three testing times.

	No-separation	Morphological- separation	Non-morphological separation (disruption of the words’ morphemes)
Time 1	#getanzt#	ge#kauf#t	get#räu#mt
Time 2	#geträumt#	ge#tanz#t	gek#au#ft
Time 3	#gekauft#	ge#träum#t	get#an#zt

#### Word Reading Fluency

Fluency in reading of words was measured in three tests, which examined different levels of possible generalization effects of the trainings. These included the reading of trained words, the reading of untrained words which share morphological structures with the words appearing in training, and the reading of words in a standardized word reading fluency test.

##### Reading of trained words

Three parallel lists of words were created by sampling 291 items from the total items appearing in training. Due to time constraints, not all items presented in training could be included in this task. Therefore, randomly selected items were included from each morphological form which appeared in training (regularly inflected forms, *-s* plurals and *-t* participles; plural marker -*n* for feminine nouns; *-ung* nominalizations and -chen diminutives with and without umlauted stems). Examples of the items presented appear in Table 2 of the Supplemental Material. One list was presented at each testing time and in a counterbalanced manner across participants. Considerations for using different lists at each testing time appear under the “Procedure” section. This task was administered individually, by presenting the participants each list on a separate printed page. The lists were matched in terms of number of items (97), mean word-length (all lists had a mean length of 7.4 letters) and frequency (mean appearances in each of the three lists was: 549.25, 551.68, and 549.6, according to dlexDB, Heister et al., 2011). The lists were also matched in terms of number of items from each of the morphological forms included in training. Participants were instructed to read the words aloud as accurately and as fast as they can, and a score representing the words correctly read within one minute was calculated.

##### Reading of untrained words

The test was created by compiling three parallel word lists, comprising 97 words each (see examples in Table 2 of the Supplemental Material). One form was administered at each testing session, and in a counterbalanced manner across participants. As in the case of the reading task of trained items, this task was administered individually, by presenting the participants each list on a separate printed page, which they were required to read out aloud. The lists were matched in terms of mean word length (7.44, 7.52, and 7.63 letters in a word) and frequency (532.91, 539.37, and 538.82 appearances, according to dlexDB, Heister et al., 2011). The words in these lists comprised word-stems which did not appear in training, and pre- or suffixes which did appear in training (regularly inflected forms, *-s* plurals and *-t* participles; plural marker -*n* for feminine nouns; *-ung* nominalizations and -chen diminutives with and without umlauted stems). The number of appearances of each pre- and suffix was also matched between the lists. The instructions given to participants and the scoring procedure were the same as in the reading test of trained words.

##### Word reading in a standardized test

The subtest of word reading efficiency from the SLRT II (Moll and Landerl, 2010) was used. The test is administered individually, with its items presented on a printed page. A list of 156 items (nouns and verbs), preceded by eight example items appear on this page. The items are arranged in eight columns, ordered in an increasing level of difficulty as far as word length, frequency, and complexity (in terms of syllable structure) are concerned. The children are instructed to read as fast and as accurately as they can and are stopped after one minute. Each correct reading of a word earns one point. Parallel test reliability coefficients of this test are above 0.90. The test has two parallel forms (A and B), which allowed the use of different forms in the three session of testing. These were administered in a counterbalanced manner across participants (A, B, A or B, A, B). The results of the first testing-time were taken as a background measure, and participants achieving a score at the 30th percentile or lower were included in training.

#### Reading Fluency and Comprehension

The standardized “Leseverständnistest für Erst- bis Sechstklässler” (ELFE 1–6; Lenhard and Schneider, 2006) was administered. As this test examines reading comprehension under time constraints, this measure represents both fluency in reading and comprehension. The test comprises three subtests: (a) word comprehension, (b) sentence comprehension, and (c) text comprehension. In the first subtest children have to choose the name of an object presented as a picture out of four written words (72 words altogether). In the second subtest, including 28 items, the children are required to choose a word matching the context of a written sentence out of four written words. In the third subtest, children are required to read short paragraphs and to answer multiple-choice comprehension questions (20 paragraphs altogether). The test is administered in groups in a paper and pencil form. The children are stopped after two minutes in the word and sentence subtests and after six minutes in the text subtest. Two examples precede each subtest. The children are required to read silently. Each item correctly answered earns one point. A sum score of the three parts is calculated. Cronbach’s alpha reliability coefficients of the subtests are between α = 0.92 and α = 0.97. This test offers two parallel forms (A and B). These were administered in the three sessions of testing in a counterbalanced manner across participants (A, B, A or B, A, B).

#### Spelling

As in the case of the word reading tests, three spelling tests were administered in each testing session, in order to examine the extent of possible effects of generalization. The tests included: spelling of trained words, spelling of untrained words which shared morphological structures with trained items, and a standardized spelling test. The tests were administered individually in a pencil and paper form.

##### Spelling of trained words

Three parallel lists of words were created by sampling 126 items from the total items included in training (none of the items were shared with the items presented in the word reading tasks, see examples in Table 2 of the Supplemental Material). One list was dictated in each testing session (presented in a counterbalanced manner across participants). All words imposed ambiguity in spelling of the word stems (i.e., phoneme–grapheme conversion did not suffice in order to produce their correct spelling). The lists were matched in terms of number of items (42), mean word length (7.76, 7.76, and 7.85), and frequency (612.73, 614.80, and 621.78 appearances, according to dlexDB, Heister et al., 2011). The lists included a sample of all morphological forms appearing in training, and these were equally represented across the three lists. The test was administered individually, while the experimenter read each word out aloud, and the child was asked to write the words down in a list. Each correct spelling of a word earned one point.

##### Spelling of untrained words

Three parallel word lists were compiled, including 42 items each (none of the items were shared with the items presented in the word reading tasks, see examples in Table 2 of the Supplemental Material). One list was dictated in each testing time (administered in a counterbalanced manner across participants). The lists were matched in terms of mean word length (7.56, 7.85, and 8.00) and frequency (515.56, 516.98 to 518.34 appearances, according to dlexDB, Heister et al., 2011). The words were composed of word-stems, which did not appear in training and of pre- or suffixes which did appear in training. In this test too, all items involved ambiguity in spelling of the word stems. The morphological forms were equally represented across the three lists. The procedure of administration and scoring was the same as in the spelling test comprising the trained words.

##### Spelling in a standardized test

As there was no available standardized spelling test with norms for both fifth and sixth graders at the time of administration, different aged-matched standardized tests were used for participants in the two grade levels. The two tests were administered in groups in a pencil and paper form. The “Diagnostischer Rechtschreibtest für fünfte Klassen,” DRT 5 (diagnostic of spelling for fifth graders, Grund et al., 2004) was administered to the fifth graders. According to the instructions of this test, a form is presented to the children which includes 51 written sentences, with one missing word per sentence. The experimenter reads each sentence out aloud, including the missing word, which the child is required to write on a blank line in the form. The test focuses on basic vocabulary (nouns, adjectives, and verbs) representing the most important spelling cases in the language. Split-half reliability of this test is *r* = 0.93. The sixth graders were presented with the second part of the “Rechtschreibtest für 6. und 7. Klassen” (RST, Rieder, 1992). Similarly to the DRT-5, a text which contains 29 missing words is presented to participants. An experimenter reads out aloud the text, including the missing words, and the children are asked to write down these words in the appropriate blank lines. As in the case of the spelling tests of trained and untrained items, the spelling of the target words in this test require orthographic knowledge (i.e., decoding of phonemes into graphemes is insufficient for correct spelling of these words). Internal reliability is between Cronbach’s α = 0.87 and α = 0.93. Both tests, DRT-5 and RST 6-7, have parallel forms (A and B). These were used in each testing time in a counterbalanced manner across participants (A, B, A or B, A, B). As each group tested included both fifth and sixth graders, the scores from the two tests had to be combined. This was done by converting the raw scores into percentile (PR) according to the tests’ norms.

#### Training

**FIGURE 1:**
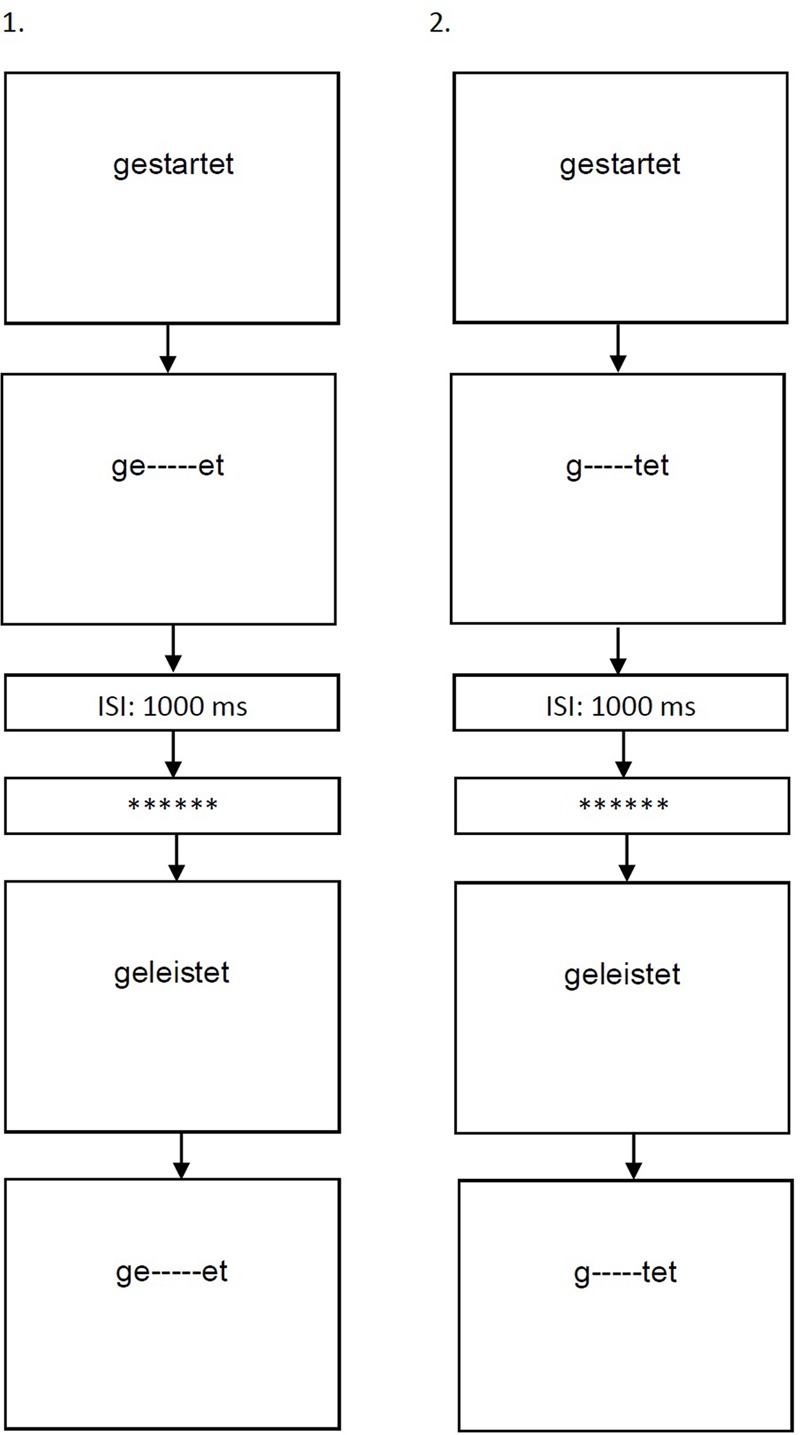
Examples of the two training programs: (1) morpheme-based training. (2) Control training. The two programs consisted of a lexical decision task, in which the presentation of an orthographic unit within a word was restricted in time- the word-stem in the morpheme-based training and a non-morphological unit in the control training. The rest of the letters in each word remained on screen until response. Each stimulus (word/pseudoword) was followed by a blank screen appearing for 1000 ms (inter-stimulus interval, ISI) and by a screen with a forward mask consisting of 6 asterisk marks appearing for 500 ms.

Two training programs were developed, in a form of a computerized visual lexical decision task, in which words and pseudoword were presented at the center of a computer screen, one after the other. The training was programed using the E-Prime software (Schneider et al., 2002). Participants were asked to decide as quickly and as accurately as they can, whether each item presented is a real word or a pseudoword (by pressing two different keys on the keyboard). Presentation of each item was terminated by response. A blank screen (appearing for 1000 ms) and a visual mask (a line of asterisks presented for 500 ms) appeared between the stimuli, in order to separate their processing. The difference between the two programs lied in the orthographic unit manipulated within each item presented (**Figure 1**):

(1) Morpheme-based training: The duration of presentation of the word-stem of each stimulus was restricted. The rest of the letters (pre- and suffixes) and small dashes replacing the letters of the word-stems, remained on screen until response (e.g., the word *gelernt*, meaning “learned,” appeared on screen, while the unit *lern* appeared for a limited time, and *ge- - - -t* remained on screen until response).
(2) Control training: As time-constraints in themselves were found to enhance reading performance (Breznitz, 2006), a control training was designed to allow isolating the possible effect of the morphological manipulation provided in the morpheme-based training. The procedure of the control training was the same as in the morpheme-based training, except that the letters manipulated did not constitute a meaningful lexical unit (e.g., the word *gelernt*, meaning “learned,” appeared on screen, while the unit *eler* appeared for a limited time, and *g- - - -nt* remained on screen until response). The non-morphological unit manipulated was equivalent in length and similar as much as possible in its position in the word to the word-stems manipulated in the morpheme-based training.

The two training programs included the same items: verbal inflections and noun derivations of various frequencies (ranging from rare to highly frequent according to the dlexDB, Heister et al., 2011). As aforementioned, there is no consensus on whether all German complex words are morphologically decomposed in reading (Clahsen, 1999; Penke, 2006; Smolka et al., 2007). Consequently, only certain classes of words were included, for which an agreement appears to exist: regularly inflected forms, *-s* plurals and *-t* participles; plural marker -*n* for feminine nouns; *-ung* nominalizations and -chen diminutives. Pseudowords were created on the basis of the real words, by changing 1–3 letters from the words’ stems. The same manipulation applied on real words in the two training conditions was also applied on pseudowords (e.g., in the case of the morpheme-based training, the pseudoword *geschernt* was presented, while the unit *schern* appeared for a limited time, and *ge- - - -t* remained on screen until response. In the control training, the duration of presentation of the unit *escher* was manipulated, while *g- - - - - -nt* remained on screen until response).

In order to take into account variation between participants in reading rate, the duration of presentation of the units manipulated in the two programs was set individually using a similar method to the one previously applied by Breznitz (2006): a lexical decision task comprising the same word classes and morphological forms presented in training was administered prior to the training. The task did not include any manipulation involving time constraints. Each stimulus was presented on screen, one after the other, while its presentation was terminated when participants decided whether the stimulus was a word or a pseudoword (by pressing one of two keys on the keyboard). Based on performance in this task, the individual per-letter reading rate was calculated. The initial duration of presentation of the manipulated units in training was set by multiplying the number of letters in the unit by the individual per-letter reading rate (e.g., the duration of presentation of the unit *Heiz* in *Heizung*, meaning “heating,” was the individual per-letter reading rate times 4). This duration was further restricted by 5% per training block, provided accuracy stayed beyond 80%. A block included 20 stimuli, half words and half pseudowords, sharing the same morphological form (see Table 3 of the Supplemental Material). Each training program included 144 blocks altogether, divided between 12 training sessions. In other words, in each training session participants responded to 240 items, 120 words and 120 pseudowords (20 items in a block × 12 blocks in a session). In order to reduce effects of re-exposure to the same items, each item appeared only once throughout the training.

As a repeated lexical decision task was expected to have an exhaustive effect on participants, other very short tasks were added between blocks, with the purpose of keeping participants interested and involved in the training sessions. The tasks included one to two questions presented after each block, such as different trivia questions, inquiry on personal views on school and learning, and questions concerning the items presented in previous blocks (e.g., did you notice any kind of animal name in the previous block?). The same questions were presented in the two training programs.

### Procedure

**FIGURE 2:**
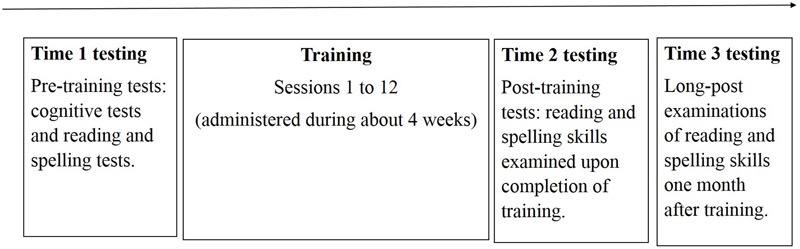
Description of the experimental procedure.

Training was carried out in small groups, while each child worked individually on a computer. Experimenters supervised the training, and verified that the tasks were understood and followed by the children. The background tests and the pre-training reading and spelling tests were administered in one meeting, which ended with the first training session. Eleven additional sessions of training followed thereafter (administered within about 4 weeks). The individually administered post-training reading and spelling tests were presented right after the last training session. The post-training tests administered in groups (ELFE 1-6, DRT 5, and RST 6-7) were presented to the children 1–2 days after the last training session. These group tests were also administered in separate days. An additional series of post-tests were administered one month after training, in order to examine whether effects were maintained (**Figure 2**). Each testing session lasted 0.75–2 hours, and a training session lasted 15–25 minutes. Participants were asked to work quickly and accurately in all tasks.

It should be noted, that as three testing sessions were planned, and within a rather short time frame, the use of the same test forms in all testing sessions, may have resulted in effects of retesting, including possible ceiling effects. In order to avoid this possibility, three parallel versions of tests were created for the tasks developed in the present study, and one of the criteria for choosing a standardized task was that it would have parallel forms. Although parallel forms may reduce effects of retesting, some influence of the different items presented in each testing time (and even when items are carefully matched) cannot be ruled out. In an attempt to reduce this possibility, the three versions of each test were presented in a counterbalanced manner across participants and testing points (for example, one participants was tested with version 1 at Time 1, with version 2 at Time 2 and with version 3 at Time 3. The next participant was tested with version 3 at Time 1, with version 1 at Time 2 and with version 2 at Time 3. Another participant was then tested with version 2 at Time 1, version 3 at Time 2, and with version 1 at Time 3).

## Results

### Performance of the Two Groups Prior to Training

It was first examined whether the two groups differed in any of the measures prior to the intervention. Performance in the background measures is presented in **Table 2**. *T*-tests for independent samples confirmed that the two groups did not differ significantly in terms of estimated IQ, vocabulary and decoding skills, as well as in age. It was further examined whether the two groups differed prior to training in performance in any of the other tasks, which were administered as pre- and post-training tests. To this end, *t*-tests for independent samples were carried out on the Time 1 measures, and these confirmed that there were no significant differences between the groups (**Table 3**, while the means and standard deviations of these tests are presented in **Tables 4–6**).

**Table 2:** Background information on the two groups of participants (mean age, non-verbal and verbal IQ, and decoding scores, standard deviations in parentheses).

	Morpheme-based	Control	*t*(45)	*P*
	training	training		
Age in years	11.24 (0.93)	11.25 (0.94)	-0.030	0.976
ZVT IQ score	98.67 (11.07)	98.04 (14.14)	0.169	0.867
Vocabulary standardized score	8.67 (2.14)	8.52 (2.64)	0.207	0.837
Decoding (items decoded correctly in 1 minute)	39.83 (11.00)	41.70 (10.66)	-0.589	0.559

**Table 3:** Comparison of performance in the reading and spelling tests at Time 1 testing between the two groups.

	*t* (43)	*p*
Word Disruption: no-separation condition	-3.67	0.715
Word Disruption: Morphological separation	-0.913	0.367
Word Disruption: Non-morphological separation	-0.644	0.523
Word Disruption: Difference between the no-separation condition and the morphological condition	0.803	0.426
Word Disruption: Difference between the no-separation condition and the non-morphological condition	-0.073	0.942
Spelling of trained words	-1.180	0.245
Spelling of untrained words	-1.014	0.316
Spelling in a standardized test	-0.594	0.556
Reading of trained words	-1.248	0.219
Reading of untrained words	-1.005	0.320
Reading of words in a standardized test	-0.716	0.478
Reading fluency and comprehension	-1.175	0.246

*The mean scores and standard deviations are presented in **Tables 4–6**.*

**Table 4:** Mean performance (words per minute, standard deviations in parentheses) of the two groups in the Word Disruption Task across the three testing times (T1, T2, T3).

	Morpheme-based training			Control training		
	T1	T2	T3	T1	T2	T3
No-separation	33.46 (11.20)	45.22 (16.68)	41.88 (12.21)	33.93 (15.20)	42.65 (15.00)	43.34 (15.03)
Morphological separation	27.99 (8.58)	41.19 (11.50)	43.69 (11.91)	30.05 (10.44)	37.01 (12.09)	39.46 (10.67)
Non-morphological separation	19.35 (5.23)	23.13 (7.38)	26.81 (7.20)	20.16 (8.05)	26.25 (7.34)	28.45 (8.34)
Difference no- separation and morphological separation	5.99 (9.67)	2.68 (8.70)	-1.83 (8.71)	2.95 (7.10)	4.10 (7.09)	3.17 (5.73)
Difference no- separation and non-morphological separation	13.75 (9.98)	21.14 (16.62)	15.49 (9.95)	13.38 (8.76)	15.21 (9.97)	15.41 (9.35)

*Time = T (1-3).*

**Table 5:** Mean performance of the two groups (standard deviations in parentheses) in the spelling tests across the three testing times (T1, T2, T3).

	Morpheme-based training			Control training		
	T1	T2	T3	T1	T2	T3
Spelling of trained words (accuracy)	16.06 (4.08)	21.17 (4.82)	20.52 (4.77)	18.02 (5.61)	20.59 (6.51)	19.61 (6.04)
Spelling of untrained words (accuracy)	19.33 (3.99)	22.17 (5.31)	24.38 (4.45)	21.09 (6.57)	22.25 (6.85)	22.77 (6.83)
Spelling in a standardized test (PR scores)	25.50 (22.79)	38.65 (26.05)	40.15 (26.21)	30.89 (27.27)	38.97 (21.50)	35.42 (26.53)

*Time = T (1-3), PR = Percentile.*

**Table 6:** Mean performance of the two groups (standard deviations in parentheses) in the reading tests across the three testing times (T1, T2, T3).

	Morpheme-based training			Control training		
	T1	T2	T3	T1	T2	T3
Reading of trained words (words per minute)	34.89 (14.57)	42.75 (17.15)	39.95 (16.85)	36.78 (13.01)	43.33 (16.01)	43.86 (19.04)
Reading of untrained words (words per minute)	35.66 (15.14)	42.60 (17.20)	39.16 (16.38)	36.46 (12.96)	43.09 (16.24)	43.11 (19.38)
Reading of words in a standardized test (words per minute)	64.88 (15.40)	71.42 (16.48)	70.08 (14.45)	66.78 (17.42)	71.83 (17.71)	73.57 (19.41)
Reading fluency and comprehension (Z scores)	-1.18 (0.42)	-0.48 (0.55)	-0.26 (0.55)	-0.98 (0.74)	-0.21 (0.81)	0.08 (0.87)

*Time = T (1-3), PR = Percentile.*

### Effects of the Manipulation Applied in the Word Disruption Task Prior to Training

In order to examine whether the manipulation applied in the Word Disruption Task had its expected effect (disruption in word reading fluency) regardless of training, we compared performance in the three conditions of presentation (morphological, non-morphological, and no-separation) prior to the interventions. A repeated measure ANOVA was carried out with performance in the three conditions of presentation at Time 1 as a within-participant factor, and group (morpheme-based training and control training) as a between participant factor. A main effect for condition of presentation was obtained [*F*(2,90) = 58.30, *p* = 0.000, $\eta_{p}^{2}$ = 0.593]. Bonferroni pair-wise comparisons indicated significant differences between the no-separation condition and the morphological condition (*p* = 0.011), between the no-separation condition and the non-morphological condition (*p* = 0.000), as well as between the morphological and the non-morphological conditions (*p* = 0.000). The means in **Table 4** confirm that the symbols created a disruption in word reading fluency when violating the sequence of letters within words (i.e., in both the non-morphological and the morphological conditions), and suggest that this effect was more pronounced in the non-morphological condition than in the morphological condition.

### Effects of the Training Programs

Next the main question of this study was addressed, i.e., what are the effects of the training programs on the different literacy skills tested. To this end, 3 × 2 repeated measure ANOVA analyses were carried out, with testing time as a within-participant factor (with three levels: Time 1, Time 2, and Time 3), and group as a between-participant factor (with two levels: morpheme-based training and control training). The results of these analyses appear in Table 1 of the Supplemental Material. Bonferroni pair-wise comparisons were applied as *post hoc* comparisons in order to test differences between the three testing times. In case a significant interaction was obtained between testing time and group, additional repeated measure ANOVA analyses were carried out separately for each group (with three levels of testing times as a within-participant factor), and Bonferroni pair-wise comparisons were, once again applied as *post hoc* comparisons. This was done in order to better understand the differences between the groups.

### Morphological Analysis in Reading

First, it was examined whether the participants improved in terms of word reading fluency within each condition of presentation in the Word Disruption Task. The 3 × 2 repeated measure ANOVAs indicated main effects for testing time in all conditions [no-separation: *F*(2,90) = 31.32, *p* = 0.000, $\eta_{p}^{2}$ = 0.416; morphological separation: *F*(2,90) = 44.65, *p* = 0.000, $\eta_{p}^{2}$ = 0.534; non-morphological separation: *F*(2,90) = 27.84, *p* = 0.000, $\eta_{p}^{2}$ = 0.388]. Bonferroni pair-wise comparisons indicated significant differences between T1 and T2 and between T1 and T3 (*p* = 0.000 in both comparisons) in all conditions of presentation. A difference between T2 and T3 was found only in the non-morphological condition (*p* = 0.026). The means in **Table 4** indicate that the two groups increased their word reading fluency in all conditions, at least from T1 to T2 and from T1 to T3. An interaction between testing time and group was obtained in the morphological condition only: *F*(2,90) = 3.29, *p* = 0.042, $\eta_{p}^{2}$ = 0.078. Further ANOVA analyses carried out separately on each group indicated that both groups improved their word reading fluency in this condition [morpheme-based training: *F*(2,46) = 35.42, *p* = 0.000, $\eta_{p}^{2}$ = 0.651, with significant differences between T1 and T2, *p* = 0.000, and T1 and T3, *p* = 0.000; control training: *F*(2,46) = 12.22, *p* = 0.000, $\eta_{p}^{2}$ = 0.379, with significant differences between T1 and T2, *p* = 0.020, and T1 and T3, *p* = 0.000). The means in **Table 4**, however, suggest a larger improvement in the group receiving the morpheme-based training.

In order to examine whether the symbols in the morphological and the non-morphological conditions interfered with word reading fluency across the three testing times, two mean difference measures of word reading fluency were calculated: the one between the no-separation condition and the morphological condition, and the other between the no-separation condition and the non-morphological condition. These were separately calculated for each testing time. Repeated measure ANOVAs with testing time as a within-participant factor and group as a between-participant factor were carried out on these measure. A main effect was found for the difference between the no-separation condition and the non-morphological condition [*F*(2,90) = 3.26, *p* = 0.044, $\eta_{p}^{2}$ = 0.077]. Bonferroni pair-wise comparisons indicated a near significant difference between T1 and T2 (*p* = 0.072). The analysis of the difference between the no-separation condition and the morphological condition indicated a main effect [*F*(2,90) = 3.31, *p* = 0.041, $\eta_{p}^{2}$ = 0.073], in addition to an interaction between testing time and group [*F*(2,90) = 3.51, *p* = 0.034, $\eta_{p}^{2}$ = 0.077]. The means in **Table 4** suggest that the morphological disruption was reduced only in the group receiving the morpheme-based training across the testing times, with no disruption at T3. Additional ANOVA analyses carried out separately on each group confirmed a significant effect of testing time only in the group receiving the morpheme-based training [*F*(2,46) = 5.47, *p* = 0.008, $\eta_{p}^{2}$ = 0.199], with Bonferroni pair-wise comparisons indicating a significant difference between T1 and T3 (*p* = 0.018).

### Access to Orthographic Representations

#### Spelling of Trained Words

The 3 × 2 repeated measure ANOVA analysis indicated a main effect for testing time [*F*(2,90) = 21.98, *p* = 0.000, $\eta_{p}^{2}$ = 0.323], together with an interaction between testing time and group [*F*(2,90) = 3.32, *p* = 0.041, $\eta_{p}^{2}$ = 0.067]. The means in **Table 5** suggest a larger improvement in the group receiving the morpheme-based training compared to the group receiving the control training. Additional ANOVA analyses carried out on the results of each group separately indicated an improvement in both groups, with possible larger effects for the group receiving the morpheme-based training. The results of the morpheme-based training were: *F*(2,90) = 22.45, *p* = 0.000, $\eta_{p}^{2}$ = 0.473, and the pair-wise comparisons indicated an improvement from T1 to T2 (*p* = 0.000) and from T1 to T3 (*p* = 0.000). The results of the group receiving the control training were: *F*(2,44) = 4.21, *p* = 0.021, $\eta_{p}^{2}$ = 0.167, and the pair-wise comparisons indicated an improvement from T1 to T2 (*p* = 0.036).

#### Spelling of Untrained Words

The 3 × 2 repeated measure ANOVA analysis indicated a main effect for testing time [*F*(2,90) = 15.17, *p* = 0.000, $\eta_{p}^{2}$ = 0.256], together with an interaction between testing time and group [*F*(2,90) = 3.74, *p* = 0.028, $\eta_{p}^{2}$ = 0.078]. The means in **Table 5** suggest an improvement in the group receiving the morpheme-based training. Additional ANOVA analyses carried out for each group separately confirmed that a significant improvement was obtained only in this group [*F*(2,46) = 24.27, *p* = 0.000, $\eta_{p}^{2}$ = 0.513], and the pair-wise comparisons indicated an improvement from T1 to T2 (*p* = 0.003), from T1 to T3 (*p* = 0.000), as well as from T2 to T3 (*p* = 0.022).

#### Spelling in a Standardize Test

The 3 × 2 repeated measure ANOVA analysis indicated a main effect for testing time [*F*(2,90) = 16.12, *p* = 0.000, $\eta_{p}^{2}$ = 0.287], together with a near significant interaction between testing time and group [*F*(2,90) = 3.01, *p* = 0.055, $\eta_{p}^{2}$ = 0.070]. The means in **Table 5** suggest an improvement in both groups, with a larger improvement in the group receiving the morpheme based training. ANOVA analyses carried out separately on the results of each group support this possibility. The results of the morpheme based training were: *F*(2,46) = 17.86, *p* = 0.000, $\eta_{p}^{2}$ = 0.448, and the pair-wise comparisons indicated an improvement from T1 to T2, *p* = 0.000, and from T1 to T3, *p* = 0.000. The results of the control training were: *F*(2,44) = 3.30, *p* = 0.048, $\eta_{p}^{2}$ = 0.155. The pair-wise comparisons did not suggest, however, any significant differences between the testing times (comparison between T1 and T2, *p* = 0.112; comparison between T1 and T3, *p* = 0.167; comparison between T2 and T3, *p* = 0.955).

### Word Reading Fluency

#### Reading of Trained Words

The 3 × 2 repeated measure ANOVA analysis indicated a main effect for testing time [*F*(2,90) = 27.66, *p* = 0.000, $\eta_{p}^{2}$ = 0.370]. The pair-wise comparisons and the means in **Table 6** suggest an improvement from T1 to T2 (*p* = 0.000) and from T1 to T3 (*p* = 0.000).

#### Reading of Untrained Words

The 3 × 2 repeated measure ANOVA analysis indicated a main effect for testing time [*F*(2,90) = 24.82, *p* = 0.000, $\eta_{p}^{2}$ = 0.350]. The pair-wise comparisons and the means in **Table 6** show an improvement from T1 to T2 (*p* = 0.000) and from T1 to T3 (*p* = 0.000).

#### SLRT II Word Reading

The 3 × 2 repeated measure ANOVA analysis indicated a main effect for testing time [*F*(2,90) = 17.30, *p* = 0.000, $\eta_{p}^{2}$ = 0.278]. The pair-wise comparisons and the means in **Table 6** point to an improvement from T1 to T2 (*p* = 0.000) and from T1 to T3 (*p* = 0.000).

### Reading Fluency and Comprehension

The 3 × 2 repeated measure ANOVA analysis indicated a main effect for testing time [*F*(2,90) = 96.81, *p* = 0.000, $\eta_{p}^{2}$ = 0.688]. The pair-wise comparisons and the means in **Table 6** suggest an improvement from T1 to T2 (*p* = 0.000), from T1 to T3 (*p* = 0.000), as well as from T2 to T3 (*p* = 0.001).

## Discussion

This study set out to examine the effects of a computerized morpheme-based training on reading and writing skills in fifth and sixth graders, who struggle with literacy skills and speak a language other than the language of instruction (German) at home. In line with the study’s predictions, the morpheme-based training contributed beyond the control training to a number of basic literacy skills (morphological analysis in word recognition and access to orthographic representations in spelling), but not to reading fluency and comprehension.

First the results of the Word Disruption Task are discussed –a task designed to examine whether morphological analysis is carried out as a process of word recognition. The comparison between the three conditions of this task at Time 1 (i.e., prior to training) confirmed that the non-orthographic symbols integrated in between the words’ letters created an interference in word reading fluency (compared to the no-separation condition). Furthermore, the results indicate that the interference was the largest when the sequence of the morphemes’ letters within words was violated (i.e., in the non-morphological condition). The present results converge with the results reported by Sonnleitner (2013), who examined younger children (second graders) to whom German was a first language. Notably, although Sonnleitner (2013) compared the violation of the morphemes’ letters in a word to the violation of another linguistic unit –syllables (in addition to a no-separation condition), the former still created the largest disruption. Together, these results confirm the role of morphological analysis in reading of German. While this role was previously demonstrated mainly in studies of adults with typical reading skills (e.g., Clahsen et al., 2003; Smolka et al., 2007), the current results suggest that regardless of training, fifth and sixth graders with poor literacy skills (and to whom German is a second language) also carry out morphological analysis of words in reading – at least to a certain extent.

The analysis examining the effects of the training programs on performance in the Word Disruption Task indicated that both groups improved in word reading fluency across the three testing times in all conditions (no-separation, morphological separation, and non-morphological separation). However, an interaction between testing time and group was found only in the morphological condition, and the results suggest a larger improvement in word reading fluency in the group receiving the morpheme-based training. This may indicate that following training, this group benefited more than the control group from the separation between words’ morphemes. An interaction between testing time and group was also obtained in the measure representing the difference in word reading fluency between the no-separation condition and the morphological condition. Further analysis suggests that the disruption caused by the symbols separating the words into their morphemes was reduced (between T1 and T3) only in the group receiving the morpheme-based training. As there was practically no disruption in word reading fluency in this group by the third testing time, morphological analysis may have been integrated into the process of word recognition (at least in the case of reading the morphological structures included in this task). These findings are in line with previous studies examining the effects of different strategies of morphological instruction on morphological knowledge and awareness (Goodwin and Ahn, 2010, 2013). However, while previous studies examined tasks involving explicit processing of morphological units (such as circling the main morpheme in morphologically complex words or specifying the meaning of word parts, see Bowers and Kirby, 2010; Lesaux et al., 2010; Harris et al., 2011), here we applied a task, which may indicate that morphological processing improved as an implicit procedure of word processing.

A main effect for testing time was also found in the measure representing the difference between the no-separation condition and the non-morphological condition, while the Bonferroni pair-wise comparisons indicated that the interference in word reading fluency tended to increase from T1 to T2. These results suggest that as word reading fluency improved in both groups, the non-morphological separation condition interfered with fluency in reading to a larger extent. Although there was no significant interaction between testing time and group in this difference measure, the means suggest that the interference increased mainly in the group receiving the morpheme-based training (from T1 to T2). As this group was trained to quickly locate the core morphological units of words, the violation of the sequence of letters within morphemes in the non-morphological condition may have interfered with the process of word recognition of this group in particular.

Next spelling is discussed. The ANOVA analysis of spelling of trained items showed an interaction between testing time and group. The means in **Table 5** and the ANOVAs carried out on each group separately indicate that both groups improved in spelling of trained item, and suggest a larger improvement, which also extended to the third testing time, in the group receiving the morpheme-based training. As each target-word was presented only once in training, a single presentation may have sufficed in order to induce some improvement in the two groups, while the morphological manipulation used in the morpheme-based training appeared to have had an added value.

Two levels of effects of generalization were further tested: generalization to spelling of untrained word-stems (integrated into trained pre- and suffixes) and generalization to spelling in a standardized test. The analysis of the spelling test of untrained word-stems showed an interaction between testing time and group, while the analysis carried out separately on each group indicated a significant improvement only in the group receiving the morpheme-based training and that this effect was maintained in T3. Hence, a partial effect of generalization can be concluded. The examination whether the effects of the morpheme-based training extend to the spelling of untrained morphological structures resulted in a marginal significant interaction between testing time and group in the standardized spelling test. Although the subsequent ANOVA analyses, carried out separately on each group, indicated a main effect for testing time in both groups, the *post hoc* comparisons were significant only in the group receiving the morpheme-based training (between T1 and T2 and between T1 and T3). Together, these results confirm the role of morphology in spelling development in the German language, as was recently put forward by Bowers and Bowers (2017) for the case of English (also see Bangs and Binder, 2016; Fracasso et al., 2016). The question arises, however, how participants receiving the morpheme-based training improved in spelling of items they were not exposed to in training. As morphological analysis was suggested to have an important role in lexical access (Rastle et al., 2000; Marslen-Wilson et al., 2008), the training of this process may have led to a successful search for orthographic representations in the mental lexicon, which in turn reduced arbitrariness in spelling. Another possibility is that the repeated deletion of the word-stems directed the attention of the trainees to the modularity of morphologically complex words. Being aware that words are composed of morphemes shared by other words, which may have been familiar to the trainees in their written form, could have helped them in producing correct spellings using analogies (Elbro and Arnbak, 1996). Good et al. (2015) also recently demonstrated positive effects of an explicit morphological awareness training on spelling and vocabulary, and these effects extended to untrained words.

The improvement in spelling following the morpheme-based training is in line with the results of the pilot study, which examined the same training technique in readers of Hebrew (Bar-Kochva, 2016). In this examination, a very short termed training was provided (∼50 minutes of training divided between two sessions), and the results suggested some advantage of the morpheme-based training over the control training in spelling of untrained items (while both groups improved in spelling of trained items). These results imply that a morpheme-based training, which imposes a demand of morphological analysis in word recognition, contributes to spelling skills in languages with distinctively different characteristics. The role of morphology in improving spelling skills in various languages and orthographies gets further support from studies examining the effects of explicit morphological instruction in additional languages (e.g., see Elbro and Arnbak, 1996 for Danish; Tsesmeli and Seymour, 2009 for English; and Taha and Saiegh-Haddad, 2016 for Arabic).

The ANOVA analyses of the measures of reading fluency and comprehension indicated a main effect for testing time, with no interaction between testing time and group. The means in **Table 6** further indicate that both groups improved across the testing times in these measures. The morpheme-based training did not then have any unique effect on performance in the reading tasks. At least as far as fluency in single word reading is concerned, the findings were surprising, as the training of morphological analysis in reading was expected to enhance lexical access, thereby leading to quicker word recognition skills. At the same time, the pilot study of the same morpheme-based training examined in Hebrew readers also did not result in any unique effect on measures of reading fluency and comprehension (Bar-Kochva, 2016). Similar findings referring to reading fluency and comprehension were reported in the meta-analysis examining the effects of other morphological interventions (Goodwin and Ahn, 2013). Fluency in reading was suggested to be based on accuracy and automaticity in the various aspects contributing to it, including integration of phonological, orthographic, semantic, syntactic, and morphological processes (Wolf and Katzir-Cohen, 2001). With this definition in mind, training morphological skills alone may not suffice in order to produce noticeable gains in such a complex process –even not in the case of fluency in single word recognition, and all the more so in the most complex process of reading –i.e., comprehension.

Yet the lack of effect of the morphological training on reading comprehension requires further consideration, in view of correlations reported between morphological skills and reading comprehension (e.g., Deacon et al., 2014, 2017). In addition, some positive effects of morphological interventions on comprehension were previously reported, although these reports are inconsistent (Bowers et al., 2010; Goodwin and Ahn, 2010, 2013; Lyster et al., 2016). Variance in the type of trainings provided in different studies may explain, at least in part the conflicting results. Some of the studies reported interventions focusing on morphological training alone, while others provided morphological training as part of a more comprehensive program, which addressed also other aspects of literacy skills, such as vocabulary (see Goodwin and Ahn, 2013 for a review). As would be expected, Goodwin and Ahn (2010) found that morphological interventions provided as a part of comprehensive instruction programs were more effective at improving children’s reading achievement than interventions with an exclusive focus on morphology. In their analysis, Goodwin and Ahn (2013) also found that one of the factors moderating the effect of morphological interventions on comprehension was the length of the training, with trainings including above 20 hours producing significant gains. In the present study, however, a considerable shorter training was provided.

Three possible factors, which were shared by the two groups (and cannot be disentangled in this study), may then explain the gains obtained in reading fluency and comprehension. These are re-testing, involvement in reading tasks and the imposing of time constraints on reading. Of these factors, the involvement of time constraints in the two trainings deserves further attention. This factor has been repeatedly shown to have a positive impact on fluency in reading and under certain conditions –on comprehension (Breznitz, 2006; Breznitz et al., 2013; Nagler et al., 2014). It may further be mentioned that a recent study suggests that time constraints have a positive effect on fluency in reading, regardless of whether morphemes were manipulated in the presentation of texts or not (Bar-Kochva and Hasselhorn, 2015). In this study, texts were presented on a computer screen, while the duration of presentation of different orthographic units was restricted by deleting the texts from screen in the direction of reading. There were four experimental conditions of text deletion: the texts were deleted one letters after the other, morpheme by morpheme, word by word, or arbitrary orthographic units were erased one after the other. All these conditions of presentation produced positive and similar effects on reading fluency (compared to a reading condition which did not include any manipulation on the presentation of texts). This study, in addition to the present one, converge to suggest that morphological manipulations applied on the presentation of words or texts may not have an effect on measures of reading fluency and comprehension beyond the effect of time constraints.

Practical implications of the current results are further considered. The analysis of the Word Disruption Task at Time 1 suggests that the skill of morphological analysis in reading was available to the children prior to training, at least to some extent. Nonetheless, the further training of the same skill produced positive effects on spelling. These results stand well in line with studies of other populations, which indicate that morphological skills are both available to participants who struggle with literacy skills, and should be further addressed as a means of promoting literacy acquisition. Quémart and Casalis (2017), for example, have shown that French speaking children (ages 10–15) with dyslexia benefited from certain morphological structures of words in spelling. Results by Cavalli et al. (2017) who examined university students with dyslexia, also suggested that morphological skills may serve as a compensating skill in dyslexia. As far as morphological training is concerned, Taha and Saiegh-Haddad (2016) showed that poor readers of Arabic responded more strongly to a morphological intervention than skilled readers. Together, these results highlight the educational relevance of this type of intervention in enhancing literacy skills of students of various populations. At the same time, the current results also point out that morphological training should be integrated in a more comprehensive program addressing additional literacy-related skills in trying to promote fluency in reading and comprehension. An additional practical aspect of the present study lies in the type of intervention provided. The results indicate that a computerized program, which addresses implicit morphological analysis in reading is an effective training method. Considering that explicit teaching of morphological knowledge often requires more resources (e.g., a tutor), this method may offer a more economical intervention.

This study has several limitations. First, the Word Disruption Task included only words sharing the same morphological structures (pre-and suffixes) as the words appearing in training. Therefore, this task does not allow concluding whether the improvement found in morphological decomposition following the morpheme-based training extends to untrained morphological structures. Second, the trainings were designed in the form of an experimental task, with the aim of isolating as much as possible the effect of the morphological manipulation. Consequently, the trainings did not include elements expected to enhance motivation, as this factor in itself may have an influence on performance. When considering the application of the training procedure offered here in field work, methods enhancing students’ involvement in the tasks should be considered (e.g., integrating the morphological manipulation as part of a game). Third, the current study examined a group of children struggling with literacy skills, who also have a migration background. The question whether the program tested here is also relevant to children with a developmental reading disability (and who do not have a migration background) remains open. Fourth, the study included several tasks, which were designed for this study, and these were examined in a rather small sample. A larger sample, in addition to the examination of these tasks in a group of typical readers (with or without a migration background) would have provided stronger results. Fifth, in the present study we referred to the aspect of the language spoken at home as an inclusion criterion, as this aspect of migration background was suggested to be related to poor literacy skills (OECD, 2001, 2003). It should be kept in mind, however, that having a migration background involves many other aspects, which may also have an influence on literacy performance, such as different socio-economic factors which were not monitored in this study. The fact that the current sample also included children speaking different languages at home, and that they or their parents immigrated to Germany at different ages, should also be taken into account in considering the generalization of the current results. Finally, although the morphological training presented here offers the practical benefit over explicit instruction methods of not depending on the mediation of a tutor, the question whether different methods of morphological intervention also have different effects on literacy performance requires direct comparisons between such methods.

## Conclusion

The results indicate that participants receiving the morpheme-based training improved the ability to analyze words into their constituting morphemes, and suggest that they integrated this process into their word recognition routine (at least in reading the morphological structures trained). The results also indicate an improvement in the ability to access word representations in spelling, and these effects extended to untrained material. At the same time, there was no benefit of the morpheme-based training over the control training in measures of reading fluency and comprehension.

## Author Contributions

IB-K planned and carried out the study, analyzed the data and wrote the manuscript. MH supervised the study.

## Conflict of Interest Statement

The authors declare that the research was conducted in the absence of any commercial or financial relationships that could be construed as a potential conflict of interest.
