# Supplementary material for: The Training of Morphological Decomposition in Word Processing and Its Effects on Literacy Skills
Source: Front Psychol. 2017 Oct 31;8:1583. doi: 10.3389/fpsyg.2017.01583 (PMC5671569; doi:10.3389/fpsyg.2017.01583)
Supplement: Supplementary file 1 [file Table_1.pdf]

Supplemental Material

Table 1. Results of the repeated measure ANOVA analyses of the different tasks administered in the three testing times (T1, T2, T3)

	**Testing time**			**Group**			**Group X Testing time**		
	F(2,90)	*p*	η2p	*F(1,45)*	*p*	η2p	*F(2,90)*	*p*	η2p
**Morphological analysis in reading**									
No separation (words per minute)	31.32	.000	.416	.003	.957	.000	1.12	.331	.025
Morphological separation (words per minute)	44.65	.000	.534	.50	.485	.013	3.29	.042	.078
Non-morphological separation (words per minute)	27.84	.000	.388	1.09	.301	.024	.60	.551	.013
Difference no separation and morphological separation (words per minute)	3.31	.041	.073	.46	.501	.011	3.51	.034	.077
Difference no separation and non-morphological separation (words per minute)	3.26	.044	.077	.622	.435	.016	1.66	.197	.041
**Access to orthographic representations**									
Spelling of trained words (accuracy)	21.98	.000	.323	.01	.908	.000	3.32	.041	.067
Spelling of untrained words (accuracy)	15.17	.000	.256	.00	.959	.000	3.74	.028	.078
Spelling in a standardized test (PR scores)	16.12	.000	.287	.00	.965	.000	3.01	.055	.070
**Word reading fluency**									
Trained items (words per minute)	27.66	.000	.370	.23	.637	.005	1.29	.281	.027
Untrained items (words per minute)	24.82	.000	.350	.14	.706	.003	1.83	.166	.038
SLRT (words per minute)	17.30	.000	.278	.17	.685	.004	.88	.418	.019
**Fluency and comprehension**									
ELFE 1-6 (Z scores)	96.81	.000	.688	2.31	.136	.050	.52	.599	.012

Note. Time=T (1-3), PR=Percentile
