# Supplementary material for: The Training of Morphological Decomposition in Word Processing and Its Effects on Literacy Skills
Source: Front Psychol. 2017 Oct 31;8:1583. doi: 10.3389/fpsyg.2017.01583 (PMC5671569; doi:10.3389/fpsyg.2017.01583)
Supplement: Supplementary file 2 [file Table_2.pdf]

Supplemental Material

Table 2. Examples of items presented to one participant in the word reading and spelling tasks of trained and untrained items across the three testing times.

**Task**	**Morphological form**	**Testing time**		
		**T1**	**T2**	**T3**
**Reading of trained items**	-*t* participles	gegründet	gebraucht	geleistet
	-*ung* nominalizations	Schaffung	Handlung	Forschung
	plural marker –*n* for feminine nouns	Raten	Hexen	Zungen
	-*s* plurals	Hobbys	Models	Zebras
	-chen diminutives	Völkchen	Hörnchen	Kännchen
**Reading of untrained items**	-*t* participles	geleitet	geöffnet	geändert
	-*ung* nominalizations	Fütterung	Packung	Brandung
	plural marker –*n* for feminine nouns	Masken	Tauben	Puppen
	-*s* plurals	Puddings	Gorillas	Flamingos
	-chen diminutives	Plätzchen	Blättchen	Brötchen
**Spelling of trained items**	-*t* participles	gestützt	gerettet	gefolgt
	-*ung* nominalizations	Spannung	Dämmerung	Stimmung
	plural marker –*n* for feminine nouns	Flaggen	Flossen	Flotten
	-*s* plurals	Shampoos	Comics	Gullys
	-chen diminutives	Stühlchen	Glöckchen	Schäfchen
**Spelling of untrained items**	-*t* participles	gejagt	getanzt	gesiegt
	-*ung* nominalizations	Deckung	Siedlung	Reinigung
	plural marker –*n* for feminine nouns	Wetten	Dielen	Tassen
	-*s* plurals	Kommas	Extras	Tipps
	-chen diminutives	Briefchen	Liedchen	Spielchen

Note. The order of administration of items was counter balanced across participants and testing times.
