# Supplementary material for: The Training of Morphological Decomposition in Word Processing and Its Effects on Literacy Skills
Source: Front Psychol. 2017 Oct 31;8:1583. doi: 10.3389/fpsyg.2017.01583 (PMC5671569; doi:10.3389/fpsyg.2017.01583)
Supplement: Supplementary file 3 [file Table_3.pdf]

Supplemental Material

Table 3. Examples of words and pseudowords presented in different blocks of a training session.

	**Morphological form**	**Item**	
		**Words**	**Pseudowords**
**Block 1**	-*t* participles	gejubelt	gebaldet
		gelernt	getacht
**Block 2**	-*ung* nominalizations	Rechnung	Walfung
		Hoffnung	Kensung
**Block 3**	plural marker –*n* for feminine nouns	Küsten	Zokten
		Linsen	Binzen
**Block 4**	-*s* plurals	Mangos	Polls
		Echos	Katos
	-chen diminutives	Bildchen	Botzchen
		Kleidchen	Kirtchen

Note. The manipulation applied on each item presented in the two training programs appears in Figure 1.
